# Comunicaciones XLVIII Reunión de la Sociedad de la Sociedad de Neurofisiología Clínica de las Comunidades de Valencia y Murcia

**DOI:** 10.31083/RN43866

**Published:** 2025-09-24

**1**.

**Cronica de una Neoplasia Detectada**

Yadira Muñoz Alarcón^1^, Teresa Gavila Lattur^1^, Magdalena García Navarro^1^, Ana Gracia Milán^1^

^1^Servicio de Neurofisiología Clínica, Hospital San Juan, 03550 San Juan de Alicante, Alicante, España

**Introducción**: La plexopatía braquial puede ser el resultado de una invasión tumoral local, los tumores de Pancoast del pulmón pueden extenderse e invadir el plexo directamente. De forma característica, la plexopatía braquial neoplásica da lugar a un síndrome lentamente progresivo a menudo asociado a dolor prominente. En algunos casos puede ser difícil o imposible distinguir clínicamente estas lesiones de lesiones más proximales de las raíces cervicales. Los estudios de conducción nerviosa y de EMG de aguja suelen ser muy útiles para diferenciar lesiones del plexo braquial de lesiones de las raíces cervicales en estos casos. **Material y Métodos**: Se describe el caso de un paciente varón de 54 años, remitido desde atención primaria por dolor en brazo izquierdo de semanas de evolución, parestesias en cara medial del antebrazo y mano izquierda. **Resultados**: EMG: proximal de tipo axonal del del tronco inferior del plexo braquial izquierdo (mayor afectación de cordón medial), con leve afectación de fibras sensitivas y mayor afectación motora, sin que se detecte signos de denervación aguda activa en el estudio actual. Dados los hallazgos electrofisiológicos y la clínica marcada de dolor pectoral y braquial muy intenso se recomienda realizar prueba de imagen Rx tórax para descartar tumor de Pancoast. Rx tórax: Masa en Lóbulo superior Izquierdo de aprox 9 × 7 cm. TAC: Masa pulmonar en lóbulo superior izquierdo, tumor de Pancoast, con infiltración de 1^a^ costilla, pared torácica, arteria subclavia y pleura mediastínica. **Conclusiones**: Aunque los estudios de electrofisiológicos en los tumores de Pancoast, son útiles para definir la relación entre el tumor y el plexo braquial, en el caso cuestión han aportado además, información relevante para acotar el diagnóstico diferencial, favoreciendo el diagnóstico del mismo y evitando el retraso diagnóstico asociado, tan común en este tipo de tumor.

**2**.

**Bloqueo Total**

Eugenio Barona Giménez^1^, Estefanía García Luna^1^, Diana Peñalver Espinosa^1^, Marina Villamor Villarino^1^, Andrea Miró Andreu^1^, Francisco de Asís Biec Alemán^1^

^1^Servicio de Neurofisiología Clínica, Hospital General Universitario Reina Sofía, 30003 Murcia, España

**Introducción**: Un correcto estudio de electrodiagnóstico siempre debe ir precedido de una buena anamnesis y exploración física. **Material y Métodos**: Varón de 60 años con antecedente de fractura de maléolo externo en pie derecho, con intervención posterior bajo anestesia epidural con bloqueo ciático en fosa poplítea. Remitido al laboratorio de electrodiagnóstico para descartar afectación del nervio ciático poplíteo externo. El paciente refiere cuadro de paresia del miembro inferior derecho con sensación de acorchamiento global del pie. La exploración física muestra ausencia de reflejo osteotendinoso aquileo derecho, con paresia 4/5 de dorsiflexión del pie derecho y de flexores de los dedos con hipoestesia global del pie. **Resultados y Conclusiones**: Se realizó estudio electroneurográfico y electromiográfico, donde se observaron fibrilaciones y ondas positivas en los músculos gemelo medial, flexor corto del primer dedo del pie y tibial anterior derechos, como una pérdida de unidades motoras en los mismos músculos. El estudio de conducciones evidenció una ausencia del potencial de acción nervioso sensitivo de los nervios peroneal superficial y sural derechos y una asimetría de amplitud en el potenciales de acción musculares compuestos entre los nervios peroneal común y tibial posterior derechos e izquierdos. Los hallazgos obtenidos fueron congruentes con la existencia de axonotmesis parcial severa del nervio ciático común en la rodilla, en estadio subagudo de evolución. Este caso pone de manifiesto, la importancia de una correcta anamnesis y exploración física, ya que con el antecedente del bloqueo anestésico en el hueco poplíteo y los resultados de la exploración física el diagnostico quedo muy orientado.

**3**.

**Síndrome del Túnel Tarsiano Anterior. A Propósito de dos Casos**

Marina Villamor Villarino^1^, Eugenio Barona Giménez^1^, Estefanía García Luna^1^, Diana Peñalver Espinosa^1^, Francisco Biec Alemán^1^, Andrea Miró Andreu^1^

^1^Servicio de Neurofisiología Clínica, Hospital General Universitario Reina Sofía, 30003 Murcia, España

**Introducción**: El síndrome del túnel tarsiano anterior (STTA) es producido por un atrapamiento del nervio peroneo profundo (NPP) en la cara anterior del tobillo o en el dorso del pie. El NPP es un nervio mixto. Inerva los músculos del compartimento anterior de la pierna y pedio, y proporciona sensibilidad cutánea y articular a porciones del dorso del pie. Se origina a partir del nervio peroneo común (NPC) a nivel de la rodilla, desciende por la parte anterior de la pierna, y pasa por el tobillo hacia el pie por debajo del retináculo extensor, donde se bifurca en dos ramas: la rama lateral, que inerva el músculo pedio y la rama medial, que recoge la sensibilidad del primer espacio interdigital. **Material y Métodos**: Presentamos una paciente de 52 años con debilidad en pie izquierdo y otra de 58 años con hipoestesia en primer espacio interdigital de pie derecho y amiotrofia de músculo pedio. Realizamos electroneurografía sensitiva de NPP bilateral, electroneurografía motora de NPC bilateral, y electromiografía de músculos pedio y tibial anterior (izquierdos y derechos respectivamente). **Resultados**: En ambas pacientes hallamos incremento de latencia distal motora y asimetría de amplitud del potencial motor del NPC (izquierdo con respecto al derecho en la primera y derecho con respecto al izquierdo en la segunda), así como actividad espontánea en reposo y pérdida de unidades motoras con cambios neurógenos en la morfología de los potenciales de unidad motora en músculo pedio (izquierdo en la primera y derecho en la segunda). En la última encontramos ausencia de potencial sensitivo del NPP bilateralmente. **Conclusiones**: Es posible una variedad de presentaciones clínicas, dependiendo de si se comprime el NPP, su rama terminal lateral o su rama terminal medial. Hablamos de STTA parcial o incompleto, cuando sólo la rama motora o sensitiva del NPP se comprime.

**4**.

**Encefalitis Herpética. A Propósito de un Caso**

Carla Andrea Artacho Pérez^1^, Emilio González García^1^, Maria José Ortiz Muñoz^1^, Andrea Victoria Arciniegas Villanueva^1^, Jeimmy Mabel Pinzón Martínez^1^, Araceli Isabel Soucase García^1^

^1^Servicio de Neurofisiología Clínica, Hospital de Manises, 46940 Manises, Valencia, España

**Introducción**: La encefalitis herpética se caracteriza por la inflamación cerebral debido a la infección directa del parénquima cerebral por los virus del herpes simple tipo 1 o 2 (EVHS), su incidencia se estima en 1:250.000 y tasa de mortalidad del 6 al 10%, que puede aumentar al 70% sin un tratamiento adecuado. El Electroencefalograma (EEG) es una herramienta importante en el diagnóstico y control de la progresión de la enfermedad, en el que se observa lentificación difusa de la actividad basal con la pérdida del ritmo dominante posterior y grafoelementos distintivos; patrón de descargas epileptiformes lateralizadas periódicas (PLED) que, junto a la presencia de actividad crítica en ausencia/presencia de cambios clínicos y reactividad/arreactividad no clara a estímulos, se pueden establecer como signos de mal pronóstico. **Material y Métodos**: Presentamos el caso de un paciente de 75 años de edad con EVHS complicada por la progresión rápidamente agresiva y la alteración de consciencia profunda y señalamos la aportación que puede suponer la realización de EEGs seriados, periódicos y frecuentes, en el seguimiento, manejo y pronóstico de enfermos con EVHS. **Resultados**: Se realizaron 5 EEGs seriados donde se evidenció actividad basal difusa lentificada (tras varios días de suspensión de sedación), ausencia de reactividad en 3 de 5 registros, PLED temporoparietales derechas y un episodio de carácter crítico focal electroclínico con focalidad derecha y con desvío de la mirada a la izquierda de 15–20 segundos de duración que yuguló con propofol intravenoso. **Conclusiones**: Los resultados de nuestro estudio se podrían sumar al reciente cuerpo de evidencia que destaca el papel pronóstico de la reactividad en el EEG y la crucial importancia para el diagnóstico práctico de la encefalitis por herpes simple y sus complicaciones, en particular las convulsiones, el status epiléptico y las PLEDs, indicadores necrosis del parénquima cerebral.

**5**.

**La Tormenta Sin Rayos**

Estefanía García Luna^1^, Diana Peñalver Espinosa^1^, Marina Villamor Villarino^1^, Andrea Miró Andreu^1^, Eugenio Barona Giménez^1^, Roberto López Bernabé^1^

^1^Servicio de Neurofisiología Clínica, Hospital General Universitario Reina Sofía, 30003 Murcia, España

**Introducción**: El Trastorno Paroxístico No Epiléptico (TPNE) se caracteriza por actividad motora recurrente de inicio y fin brusco, duración de segundos-minutos y recuperación inmediata, que suele ir acompañada diferentes síntomas somáticos. **Material y Métodos**: Paciente varón de 18 años con antecedentes personales de malformación Chiari tipo I intervenida, es ingresado por presentar movimientos cefálicos y de miembros superiores anormales desde septiembre 2023, que han aumentado progresivamente en intensidad. En tratamiento con valproato sin mejoría y limitación de la calidad de vida. **Resultados**: Exploración física que muestra movimientos de lateralización y flexión cefálica, flexión y aducción de miembros superiores, resto normal. Resonancia magnética cerebral con cambios postquirúrgicos, sin otros hallazgos. Se realiza Electroencefalograma estándar, limitado por abundante artefacto muscular, donde se registran descargas generalizadas de actividad epileptiforme que se acompañan clínicamente de movimientos cefálicos y de miembros superiores, considerando que dichos hallazgos son sugestivos de epilepsia mioclónica juvenil. Se inicia tratamiento con valproato intravenoso sin mejoría, por lo que se decide realizar estudio Polisomnografía nocturna vigilada con monitorización, incluyendo períodos de vigilia. Se registran 45 fenómenos motores paroxísticos, con lateralización cefálica y contracción de miembros superiores durante la vigilia, y 11 episodios similares durante el descanso nocturno, que coinciden con arousals espontáneos, sin presentar un claro correlato eléctrico que sugiera origen cortical. Dichos hallazgos son sugestivos de trastorno paroxístico no epiléptico (TPNE). Ante esta situación se continúa tratamiento con placebo, quedando progresivamente libre de episodios tras 5 días, siendo dado de alta previa valoración de Psiquiatría. **Conclusiones**: El registro del vídeo-electroencefalograma es la herramienta fundamental para el diagnóstico diferencial entre trastorno paroxístico no epiléptico y sospecha de crisis con origen comicial, ya que nos permite valorar la existencia o no de correlato clínico en el registro audio-vídeo, y el correcto tratamiento farmacológico según el diagnóstico etiológico.

**6**.

**Enfermedad de Creutzfeldt-Jakob (ECJ) y Eeg, a Propósito de Tres Casos**

Julián Vázquez Lorenzo^1^, Sofía Ortigosa Gómez^1^, Sara Giménez Roca^1^, Pilar Martínez Martínez^1^, Victor Rubio Suárez^1^, Reetika Baharani Baharani^1^

^1^Servicio de Neurofisiología Clínica, Hospital Universitario Virgen de la Arrixaca, 30120 Murcia, España

**Introducción**: La enfermedad de enfermedad de creutzfeldt-jakob (ECJ) es una enfermedad neurodegenerativa fatal causada por un plegamiento inadecuado de la proteína priónica. El diagnóstico definitivo de esta enfermedad es anatomopatológico, sin embargo, los datos clínicos y pruebas complementarias Electroencefalograma, Resonancia magnética cerebral, estudio del líquido cefalorraquídeo, permiten establecer un diagnóstico probable en vida. **Material y Métodos**: Se obtienen datos de 3 pacientes a través del servicio de codificación del HUVA. **Resultados**: Se describen tres pacientes (dos mujeres y un varón) con diagnóstico de ECJ probable y edades de 66, 78 y 75 años. Todos consultan por cuadro de deterioro cognitivo subagudo rápidamente progresivo, asociando alteraciones visuoespaciales francas en dos de los pacientes y ataxia y signos piramidales/extrapiramidales en todos. Dos de ellos presentaron mioclonías clínicas y todos evolucionaron a un mutismo acinético y fallecimiento final entre los 3–8 meses desde el inicio de la clínica. Se observaron alteraciones específicas en RM en dos de los pacientes. El análisis de la proteína 14-3-3 fue positivo en todos. Se realizaron EEG secuenciales en dos pacientes. En todos los casos el EEG fue patológico, si bien sólo mostró los típicos patrones periódicos de ondas bi/trifásicas en dos de los pacientes. No se registró el patrón cíclico alternante en ningún paciente, probablemente por falta de realización de v-EEG en las fases finales de la enfermedad. **Discusión**: El v-EEG en paciente con demencia rápidamente progresiva con sospecha de ECJ es de gran ayuda en el diagnóstico premorten. Se pueden observar desde hallazgos inespecíficos (lentificación difusa, GRDA de predominio frontal) hasta hallazgos más específicos como actividades generalizadas periódicas de corta duración o LPDs. Estas alteraciones no se encuentran en todas las variantes de ECJ, y dependen del estadío de la enfermedad, por lo que su ausencia no descarta enfermedad y la realización de v-EEG seriados aumenta la sensibilidad de la prueba. **Conclusiones**: Los estudios vídeo-EEG seriados tienen especial relevancia en la ECJ, ya que forman parte de los criterios diagnósticos de la enfermedad. Describimos los casos de tres pacientes con ECJ probable esporádica.

**7**.

**Diagnóstico Diferencial del Electrorretinograma Negativo a Través de Tres Casos Clínicos**

Reetika Mukesh Baharani Baharani^1^, Patricia Vázquez Alarcón^1^, Sara Giménez Roca^1^, Víctor Hugo Rubio Suárez^1^, Pilar Rosario Martínez Martínez^1^, Julián Vázquez Lorenzo^1^

^1^Servicio de Neurofisiología Clínica, Hospital Clínico Universitario Virgen de la Arrixaca, 30120 Murcia, España

**Introduccioń**: La respuesta negativa del electrorretinograma (ERG) de campo completo se define como una disminucioń de la onda b con conservacioń de la onda a (ratio b/a <1) en la respuesta combinada de conos y bastones. La presencia de este patroń denota una alteraciońen las ceĺulas bipolares, las ceĺulas de Mul̈ler o la transmisioń del estiḿulo fotorreceptor-ceĺulas bipolares, con conservacioń de la funcioń de fotorreceptores. Material y met́odos. Se exponen tres casos clińicos de pacientes estudiados en el Servicio de Neurofisiologiá Clińica del Hospital Clińico Universitario Virgen de la Arrixaca con un patroń ERG negativo. **Resultados**: Los dos primeros pacientes son varones de 2 y 9 an os con nistagmus y miopiá magna desde el nacimiento. Ambos fueron diagnosticados mediante un estudio genet́ico de Enfermedad de las Islas de Aland y se les realizoún ERG con registro mediante electrodos de superficie infraorbitarios, en el que se observoún patroń ERG negativo bilateral. El tercer paciente, es un adulto de 65 an os con peŕdida de agudeza visual unilateral de un an o de evolucioń al que se realizoún ERG, en este caso con registro mediante electrodo corneal, obteniendo un patroń ERG negativo unilateral de probable origen vascular. **Conclusiones**: El ERG negativo es de gran utilidad en el diagnośtico diferencial de enfermedades de la retina, pudiendo encontrarse de forma bilateral y simet́rica en diferentes enfermedades hereditarias o de forma unilateral (o bilateral asimet́rica) en enfermedades adquiridas.

**8**.

**Estatus Epiléptico No Convulsivo en Contexto de Meningitis por Otitis Media. A Propósito de un Caso**

Ana María Muñoz Mateo^1^, Cristina Ipiéns Escuer^1^, Carla Blanco Pino^1^, Andrés Salas Redondo^1^, Celio Martínez Ramírez^1^, Francisco Javier Puertas Cuesta^1^

^1^Servicio de Neurofisiología Clínica, Hospital Universitario La Ribera, 46600 Alzira, España

**Introducción**: Las meningitis bacterianas agudas (MBA) pueden causar complicaciones sistémicas y a nivel neurológico. S.Pneumoniae es la causa más frecuente de meningitis bacteriana aguda en adultos de más de 20 años. Entre los factores de riesgo típicos destacan las infecciones recurrentes del oído medio y la inmunosupresión. **Material y Métodos**: Mujer de 63 años diabética que consulta por cuadro de fiebre y secreción purulenta del oído izquierdo. Presenta rigidez de nuca y alteración del nivel de consciencia. Se realiza TAC cerebral, en el que se observa la ocupación mucosa completa del conducto auditivo externo, oído medio y celdas mastoideas izquierdas. Se establece el diagnóstico de meningitis aguda por S.Pneumoniae tras confirmación con punción lumbar y antigenuria. Ante la alteración en el nivel de consciencia se solicita EEG. **Resultados**: El EEG muestra descargas epilépticas continuas de puntas y ondas agudas de mediano-alto voltaje compatible con estatus epiléptico no convulsivo, que tras la infusión de sedoanalgesia da lugar a un trazado de brote-supresión iatrógena. Se realizan controles de EEG con persistencia del estatus, siendo necesario el aumento del tratamiento antiepiléptico para evitar el estatus epiléptico recurrente. Sin embargo, la evolución de la paciente es desfavorable. **Conclusiones**: La alteración del nivel de consciencia y cefalea son síntomas neurológicos que se producen con frecuencia en MBA. Si bien la causa puede estar relacionada con crisis epilépticas, existen algunos casos en los que el deterioro del nivel de consciencia pueda estar causado por un estatus epiléptico no convulsivo. Esto demuestra la importancia de la realización de controles con EEG en casos de deterioro de consciencia, ya que estamos ante pacientes críticos con enfermedades de elevada morbimortalidad.

**9**.

**Síndrome SILENT. A Propósito de un Caso**

Ana María Muñoz Mateo^1^, Cristina Ipiéns Escuer^1^, Carla Blanco Pino^1^, Andrés Salas Redondo^1^, Habib Halim Azzi^1^, Francisco Javier Puertas Cuesta^1^

^1^Servicio de Neurofisiología Clínica, Hospital Universitario La Ribera, 46600 Alzira, España

**Introducción**: El litio es un fármaco comúnmente utilizado en el trastorno bipolar. El rango terapéutico es muy estrecho dados los efectos tóxicos que puede producir, entre ellos secuelas neurológicas. **Material y Métodos**: Hombre de 51 años con antecedentes de trastorno bipolar acude a hospital por episodios de hipotonía y posterior cuadro de movimientos tónico-clónicos en hemicuerpo derecho, asociando disartria. Va progresando el deterioro del nivel de consciencia junto con empeoramiento del estado general. Las pruebas de imagen son normales. Los niveles de litio están ligeramente aumentados. La clínica persiste a pesar de la normalización de los niveles de litio mediante hemofiltración. Se solicita EEG ante la alteración en el nivel de consciencia. **Resultados**: En el EEG se observan descargas epilépticas difusas y semicontinuas de puntas, ondas agudas y de ondas lentas, a intervalos cortos, de mediano-alto voltaje, entremezcladas con actividad más lenta, con la aparición de un trazado en el rango de ondas theta y theta-delta tras la perfusión de sedoanalgesia. Se determina la presencia de estatus epiléptico no convulsivo. **Conclusiones**: El síndrome SILENT es un síndrome de neurotoxicidad irreversible efectuada por litio. Consiste en síntomas prolongados neuropsiquiátricos y neurológicos, debidos a la toxicidad del litio. Este cuadro se desarrolla ante niveles elevados de litio, y puede persistir a pesar de la retirada del fármaco. Los signos más frecuentes son hipertonía generalizada, hiperreflexia, temblor, mioclonías, fasciculaciones y disartria. También existen casos de hipotonía o convulsiones. En el EEG puede haber un enlentecimiento difuso pero no es raro ver estatus epiléptico no convulsivo. Finalmente, cabe destacar su dificultad diagnóstica, pues puede ser confundido clínicamente con alteraciones psiquiátricas, encefalopatías metabólicas y estados confusionales.

**10**.

**Ganglionopatía Como Manifestación Clínica de una Celiaquía. A Propósito de un Caso**

Araceli Isabel Soucase García^1^, Jeimmy Mabel Pinzón Martínez^1^, Andrea Victoria Arciniegas Villanueva^1^, Emilio González García^1^, María José Ortiz Muñoz^1^, Carla Andrea Artacho Pérez^1^

^1^Servicio de Neurofisiología Clínica, Hospital de Manises, 46940 Manises, Valencia, España

**Introducción**: Las ganglionopatías, también llamadas neuronopatías sensoriales, son afecciones selectivas, subagudas y adquiridas de las neuronas sensitivas del ganglio raquídeo dorsal. Suelen asociarse a trastornos disinmunes, paraneoplásicos y tóxicos. La celiaquía es una enfermedad prevalente caracterizada por la afectación del intestino delgado y provoca alteraciones intestinales, entre otras, y en ocasiones, presenta manifestaciones neurológicas tales como la polineuropatía. Se caracterizan por afectar a las fibras nerviosas sensitivas gruesas y por tanto, los pacientes presentan quejas sensoriales tipo hipoestesias, alteraciones en la propiocepción y la vibración típicamente asimétricas. Clínicamente, es típica la arreflexia, la alteración de la marcha temprana sin debilidad asociada y el signo de Romberg +. El diagnóstico precoz y el valor de la electromiografía es esencial, ya que la clínica sensitiva puede ser la primera expresión de una enfermedad autoinmune o una neoplasia subyacente. **Material y Métodos**: Presentamos el caso de una paciente de mediana edad con clínica de parestesias fluctuantes, inestabilidad y arreflexia. Se realizaron tres electromiografías con resultados de polineuropatía sensitiva axonal, asimétrica y de predominio en miembros superiores. **Resultados**: Electromiográficamente, la ausencia de un PANS o tres con amplitud menor al 30% (LIN) en MMSS no explicable por atrapamiento nervioso, con normalidad en la velocidad, PAMC y la normalidad en la exploración con aguja son un criterio diagnóstico. En nuestra paciente se valoró un USMAR menor de 0,71. Además de las pruebas electromiográficas y tras un exhaustivo estudio, la paciente fue diagnosticada de celiaquía y se descartó malignidad. **Conclusión**: La sospecha clínica de quejas sensitivas, inestabilidad y arreflexia, junto al estudio electromiográfico, permiten el diagnóstico temprano en las ganglionopatías. Si bien la celiaquía tiene diversas manifestaciones, la ganglionopatía no es muy común. Sin embargo, está descrita por algunos autores en pacientes similares a la nuestra.

**11**.

**Hallazgos en v-EEG en la Hiperglicinemia No Cetósica: A Propósito de un Caso**

Pilar Rosario Martínez Martínez^1^, Carmen María Garnés Sánchez^1^, Sofía Ortigosa Gómez^1^, Reetika Mukesh Baharani Baharani^1^, Víctor Hugo Rubio Suárez^1^, Julián Vázquez Lorenzo^1^

^1^Servicio de Neurofisiología Clínica, Hospital Clínico Universitario Virgen de la Arrixaca, 30120 Murcia, España

**Introducción**: La hiperglicinemia no cetósica (NKH) es un trastorno metabólico autosómico recesivo caracterizada por acumulación excesiva de glicina en los tejidos, incluido el sistema nervioso central. Su prevalencia es 1:60.000 y existen dos formas: grave y atenuada. La grave aparece en los primeros días de vida cursando con encefalopatía progresiva, hipotonía, mioclonías, hipo, convulsiones, progresión a coma y muerte por apnea central. Aquellos que sobreviven muestran un retraso del desarrollo grave y crisis refractarias. La forma atenuada de NKH cursa con evolución variable. **Material y Métodos**: Presentamos una paciente con v-EEG seriados por letargia e hipotonía a estudio en el Hospital Virgen de la Arrixaca y se revisa la bibliografía. **Resultados**: Neonato pretérmino tardío que ingresa a las 24 horas de vida en UCI-Neonatal por hipotonía severa y letargia. No crisis. En la exploración destaca dos apéndices preauriculares derechos y dedo 1–2 de ambos pies cabalgados. A nivel neurológico: letargia, ausencia de apertura ocular espontánea e incompleta al dolor, respuesta a estímulos alterada, escasa succión y severa hipotonía axial. Se solicita v-EEG observándose crisis eléctricas focales y anomalías epileptiformes multifocales, en un trazado de brote-supresión (BS), que ceden tras administración de fenobarbital. Al día siguiente, v-EEG similar, añadiéndose levetiracetam. Posteriormente, v-EEG sin crisis eléctricas, persisten anomalías epileptiformes multifocales aisladas y BS. Los análisis bioquímicos mostraron niveles elevados de glicina en sangre, orina y líquido cefalorraquídeo. RM cerebral sin hallazgos significativos. Los resultados de las pruebas orientan al diagnośtico de NHK. Estudio genético normal. Se inicia alimentación con fórmula especial y quelantes de glicina. No obstante, la paciente finalmente falleció al 12^∘^ día de vida. **Conclusiones**: En la NKH el v-EEG suele mostrar un patrón de BS con puntas multifocales que puede evolucionar a hipsarritmia. El v-EEG es una herramienta útil en la orientación diagnóstica y seguimiento de esta patología.

**12**.

**Apnea Obstructiva del Sueño y Dispositivo de Avance Mandibular. Presentación de Casos Clínicos**

Nerea Torres Caño^1^, Teresa Oviedo Montés^1^, María Roldán Gómez^1^, Alba Pastora Salazar Moya^1^, Marina García Selva^2^, Sofía Rodríguez Moroder^3^

^1^Servicio de Neurofisiología Clínica, Hospital de Gandía, 46702 Gandía, Valencia, España

^2^Clínica Oliva Dental, 46780 Oliva, Valencia, España

^3^Clínica Craneosalud, 46005 Valencia, España

**Introducción**: El dispositivo de avance mandibular (DAM) es una alternativa terapéutica eficaz y segura en pacientes roncadores o con apnea osbtructiva del sueño (AOS) leve o moderado. También está indicado en pacientes más graves que no toleren el tratamiento con CPAP o cirugía. Se trata de un dispositivo que el paciente se coloca en la boca al dormir, consiste en una férula superior y otra inferior, que mantienen la mandíbula ligeramente adelantada, con el fin de aumentar el espacio faríngeo para el paso del aire, así como aumentar el tono muscular de las paredes de la faringe y evitar la obstrucción de las vías aéreas durante el sueño. **Casos Clínicos**: Se presentan 3 casos clínicos de pacientes con DAM y su evolución. Caso 1: Paciente de 48 años con diagnóstico de AOS grave (IAH 30,6) en 2020, mala adaptación a CPAP. Se coloca DAM y se realiza nueva polisomnografía en 2022, con IAH de 12,6. Caso 2: Paciente de 65 años con diagnóstico de AOS grave (IAH 33) en 2014. Se pauta CPAP con mala tolerancia, por lo que comienza con DAM en 2017, con buena adaptación y nueva PSG con IAH de 12,4. En 2022 refiere empeoramientro y se realiza nuevo estudio con DAM con IAH de 41 con DAM. Tras una serie de adaptaciones del DAM, refiere mejoría y se realizan controles sucesivos domiciliarios con IAH de 2. Caso 3: Paciente de 50 años con retrognatia diagnosticada de AOS leve (IAH 14,3) en 2020. Se coloca DAM con mejoría de la clínica, IAH 2,4 en polisomnografía realizada en 2022. **Conclusiones**: El uso del DAM en pacientes con AOS leve o moderado tiene resultados exitosos, además reduce el IAH en los casos más graves aunque este no esté indicado como tratamiento de primera línea. Para su correcto funcionamiento y determinar el mayor beneficio para el paciente, se debe ofrecer un tratamiento personalizado y seguimiento para cada caso.

**13**.

**La Ambliopía, Posibles Hallazgos en las Pruebas Neurofisiológicas Visuales**

Sandra Liliana Beltrán Castro^1^, Maria Jesus Estarelles Marco^1^, Josep Orenga Orenga^1^, Claudia Andrés Collado^1^, Lledó Orenga Pachés^1^, Nuria Ruiz Montagud^1^

^1^Servicio de Neurofisiología Clínica, Hospital General Universitario de Castellón, 12004 Castellón, España

**Introducción**: La ambliopía es una patología con una prevalencia mundial del 6,2% y la 4^∘^ causa de ceguera monocular en niños. Consiste en una alteración del sistema visual caracterizada funcionalmente por una diferencia de al menos 2 líneas en la agudeza visual entre ambos ojos, con la mejor corrección óptica. Sus causas más frecuentes son el estrabismo y la anisometropía. **Materiales y Métodos**: Presentamos dos casos clínicos de pacientes con ambliopía: Caso 1: Paciente femenina de 68 años en tratamiento con dolquine desde hace 8 años por lupus eritematoso sistémico (LES), quien solicitan ERG mf para control del tratamiento farmacológico. Hemos encontrado una afectación del ERG mf únicamente en OD (ojo derecho) con disminución de amplitud en el área más central y ERG mf del OI normal. Caso 2: Paciente femenina de 60 años con disminución de AV en OD, de tiempo indeterminado que remiten para PEV. Obtuvimos PEV-P OD con disminución de la amplitud y aumento de la latencia de la onda P100 de forma significativa respecto al contralateral. PEV-P del OI dentro de la normalidad. **Resultados**: Los hallazgos encontrados en estos dos casos, podrían ser atribuibles a ambliopía. En el primer caso, la alteración foveal de un sólo ojo se ha descrito en ojos ambliopes, mientras que la alteración por hidrocloroquina, suele ser parafoveal y bilateral. Los hallazgos encontrados en el segundo caso, con retraso de latencia y disminución de la amplitud de la onda P100, también se han descrito en el ojo ambliope. **Conclusiones**: La ambliopía es una patología frecuente en la población, que nos puede producir alteración en diversas pruebas neurofisiológicas visuales y hay que tenerla en cuenta cuando nos derivan a un paciente ambliope por otras causas.

**14**.

**La Dificultad Para Establecer un Diagnóstico de Seguridad en Narcolepsia. A Propósito de un Caso**

Andrea Sánchez Femenía^1^, Pau Giner Bayarri^1^, Ernest Balaguer Roselló^1^, Marina Murillo Martínez^1^, David Castillo Escobar^1^, Beatriz Morcillo Escudero^1^

^1^Servicio de Neurofisiología Clínica, Hospital Universitario Doctor Peset, 46010 Valencia, España

**Introducción**: La narcolepsia es un trastorno del sueño crónico caracterizado clínicamente por somnolencia diurna excesiva que puede estar acompañada de cataplejia, parálisis del sueño y alucinaciones hipnagógicas. La prevalencia es baja, siendo considerada una enfermedad rara. En las últimas décadas, el cambio en los criterios diagnósticos ha hecho que muchos pacientes narcolépticos ya no puedan ser clasificados como tales. **Material y Métodos**: Se presenta el caso de una paciente de 51 años diagnosticada de narcolepsia tipo I con cataplejia hace 20 años, según los criterios de aquel momento. La paciente, con múltiples antecedentes psiquiátricos, inició tratamiento con oxibato sódico en 2009, mejorando su sintomatología hasta el año 2019, cuando empeora de nuevo y no responde a diferentes tratamientos. En ese momento se replantea el diagnóstico. **Resultados**: Dado que la paciente se encuentra en tratamiento con 2 antidepresivos y un antihistamínico, sin ser posible su retirada, no se puede realizar un nuevo test de latencia múltiple de sueño (TLMS). Presenta actigrafía con una higiene de sueño muy alterada (días con 2 horas de sueño y otros con más de 10 horas). Por ello, se realizó una punción lumbar para determinación de hipocretinas en líquido cefalorraquídeo, con niveles de orexina-A de 244 pg/mL, descartándose así Narcolepsia tipo I. **Conclusiones**: Ante un paciente con diagnóstico de Narcolepsia tipo I antiguo, sin mejoría tras diferentes tratamientos y comorbilidad psiquiátrica, se recomienda reevaluación del caso con valoración de actigrafía, nuevo TLMS e hipocretinas.

**15**.

**Incidencia y Descripción de la Población y Trazados con Respuesta Fotoparoxística en los Electroencefalogramas Realizados en el Hospital General Universitari de Castelló Entre los Años 2013–2023**

Lledó Orenga Pachés^1^, José Vicente Orenga Orenga^1^, Alina Denisa Ghinea^1^, Sandra Liliana Beltrán Castro^1^, Claudia Andrés Collado^1^, Nuria Ruiz Montagud^1^

^1^Servicio de Neurofisiología Clínica, Hospital General Universitari de Castelló, 12004 Castellón, España

**Introducción**: La respuesta fotoparoxística (RF) es la aparición en el electroencefalograma de descargas punta-onda, polipunta o polipunta-onda de distribución occipital, frontal o generalizada provocada por la estimulación luminosa intermitente (ELI). Estudios epidemiológicos muestran una incidencia en población general entre 1 por 50.000 a 1 por 4000. **Material y Métodos**: Análisis descriptivo retrospectivo de los electroencefalogramas realizados en nuestro servicio entre los años 2013–2023, seleccionando los que registraron RF. Se realizó ELI con luz ambiente mínima, lámpara situada a 30 cm del nasion, ojos cerrados, frecuencia de trenes de 5 segundos, intervalos de reposo de 5 segundos y frecuencia de disparo creciente desde 3 Hz a 30 Hz. Se dividieron las RF en bajas menor que alfa, medias rango alfa y altas mayor que alfa. **Resultados**: Del total de trazados se objetivaron 81 con RF, que supuso 1 de cada 220 estudios realizados. Se descartaron 13 trazados que correspondían a pacientes con más de un registro, se analizó únicamente el primero, estudiando 68. El rango de edad se encontraba entre 1,5 y 86 años con una media de 20,6. El grupo de edad predominante con un 70,6% correspondía al intervalo entre 6 y 20 años. La distribución por sexos fue masculino 35,3% y femenino 64,7%. Del total de pacientes 26 presentaban diagnóstico previo de epilepsia, siendo las más frecuentes la generalizada idiopática y la ausencia típica. Se estudiaban 31 como primera crisis. La RF predominó en frecuencias medias-altas y altas. El trazado basal de los pacientes mostró actividad epileptiforme focal en 25, actividad epileptiforme generalizada en 13, actividad epileptiforme multifocal en 2 y sin actividad epileptiforme en 28. **Conclusiones**: La incidencia de RF de nuestro estudio es baja. Predominio en sexo femenino, grupo de edad mayoritario infanto-juvenil. Frecuencias medias-altas y con diagnóstico previo de epilepsia o primera crisis.

**16**.

**Polineuropatía y Gammapatías Monoclonales: Serie de Casos**

Mar Mascarell Polache^1^, Ángeles Lloret Alcañiz^1^, Tatiana Enríquez Sague^1^, Yamaris Vera Castillo^1^, Javier Salas Jordan^1^, Paula Cases Bergon^1^

^1^Servicio de Neurofisiología Clínica, Hospital Clínico Universitario Valencia, 46010 Valencia, España

**Objetivo**: Las gammapatías monoclonales son un grupo de enfermedades caracterizadas por la proliferación de células plasmáticas que secretan una proteína monoclonal. La coexistencia de polineuropatía y gammapatía es común, sobre todo en las de tipo IGM, aunque ello no siempre implique causalidad. Conocer los hallazgos electrofisiológicos descritos asociados a los distintos tipos de neuropatías paraproteinémicas, junto a la presentación clínica y las características hematológicas, es importante para un correcto diagnóstico y tratamiento del paciente. Presentamos una serie de 16 casos con diagnóstico de gammapatía monoclonal en el momento del estudio electromiográfico (EMG), analizando si los resultados coinciden con los típicamente descritos en la literatura para cada tipo de gammapatía. **Materiales y Métodos**: Se recogen 16 casos de pacientes con gammapatía monoclonal a los que se realizó EMG en el servicio de Neurofisiología Clínica del Hospital Clínico de Valencia entre 2017 y 2024. Los datos recogidos incluyen edad y sexo del paciente, tipo de gammapatía monoclonal y fecha del diagnóstico, análisis inmunológico, coexistencia de otras patologías, presentación clínica, exploración neurológica en el momento del EMG, tratamiento, y hallazgos en el EMG y, en su caso, en los sucesivos controles. **Resultados**: De los 16 pacientes estudiados, 10 (63%) cumplieron criterios de polineuropatía, de los cuales 4 presentaban gammapatía monoclonal de significado incierto (MGUS) IgM, 3 MGUS IgG, uno MGUS IGA, uno MGUS biclonal IgA/IgG y uno mieloma múltiple Bence Jones kappa. La polineuropatía en 5 de los casos (50%) fue catalogada como sensitivo-motora desmielinizante distal simétrica (DADS), y en los otros 5 encontramos diferentes tipos de afectación (sensitiva y/o motora, axonal y/o desmielinizante). **Conclusión**: El tipo de polineuropatía más común en nuestra serie fue la DADS, que es la típicamente descrita en la literatura. Algunos pacientes presentaban comorbilidades o tratamientos que podrían influir en los resultados, actuando como factores de confusión.

**17**.

**Determinación del Cronotipo Mediante Dim-Light Melatonin Onset (DLMO) en el Abordaje Clínico de los Trastornos del Sueño**

Yamaris Vera^1^, Tatiana Enriquez^1^, Mar Mascarell^1^, Javier Salas^1^, Paula Cases^1^, Manuel de Entrambasaguas^1^

^1^Servicio de Neurofisiología Clínica, Hospital Clínico Universitario de Valencia, 46010 Valencia, España

**Introducción**: El cronotipo es la propensión individual a dormir en un horario determinado dentro de las 24 horas del ciclo sueño-vigilia. Su identificación ayuda al diagnóstico y tratamiento de distintos trastornos del sueño. Se puede inferir por medio de cuestionarios (MEQ, MCTQ) o el horario libre declarado o registrado (diarios de sueño, actigrafía). Sin embargo, estas medidas en ocasiones se contradicen, y no aclaran si el cronotipo existente tiene una base biológica o conductual. El DLMO recoge la hora en que se produce el ascenso de melatonina, cuya secreción sigue un ritmo circadiano, y ocurre unas 2 horas antes del inicio del sueño. Suele realizarse en laboratorio con condiciones extremadamente controladas. El objetivo de este estudio era valorar si es factible recoger las muestras en el domicilio, definiéndose para ello varios determinantes de calidad. **Material y Métodos**: Participaron 30 pacientes (21 hombres, 70%) de 38,3 ± 16,3 años (10–61). Según horario libre, 12 tenían cronotipo vespertino extremo (VE, 40%), 9 vespertino moderado (VM, 30%), 5 intermedio (16,7%) y 4 no valorable por síndrome de fatiga crónica o trastorno conductual, existiendo un 26% de concordancia con MEQ/rMEQ. Se entregó un kit protocolizado a los pacientes para recoger en casa 5 muestras consecutivas de saliva, cada hora. La melatonina se cuantificó mediante radioinmunoanálisis. **Resultados**: Se hicieron 32 determinaciones (repetida en 2 pacientes), que mostraron cifras en ascenso. Existió importante variabilidad interindividual, por lo que se escogió el umbral dinámico en vez del estático para calcular el DLMO. Este correspondió a la última muestra en 10 pacientes, siendo el 80% VE y el 20% VM según horario libre o MEQ/rMEQ. **Conclusiones**: El DLMO protocolizado en entorno doméstico parece una herramienta fiable para determinar el cronotipo, en especial cuando se valora junto a otras medidas. Sería deseable contar con valores de referencia.

**18**.

**Espasmos Infantiles con Evolución a Epilepsia con Crisis Mioclónicas**

Diana Peñalver Espinosa^1^, Marina Villamor Villarino^1^, Eugenio Barona Giménez^1^, Estefanía García Luna^1^, Andrea Miró Andreu^1^, Helena Alarcón Martínez^2^

^1^Servicio de Neurofisiología Clínica, Hospital General Universitario Reina Sofía, 30003 Murcia, España

^2^Servicio de Neurofisiología Clínica, Hospital Clínico Universitario Virgen de la Arrixaca, 30120 Murcia, España

**Introducción**: Los espasmos son el tipo de crisis epiléptica más prevalente durante el primer año de vida. La evolución de estas crisis es heterogénea, pudiendo manifestarse como una encefalopatía epiléptica severa con retraso en el desarrollo global o una epilepsia con correcto neurodesarrollo. **Material y Metodos**: Lactante de 6 meses con desarrollo psicomotor normal, que comenzó con episodios de hiperextensión de MMSS y cabeza en salvas de 20 segundos y de hasta 5 minutos de duración. Durante su ingreso, se realizó v-EEG observándose 10–15 descargas generalizadas en salvas asociadas a hiperextensión de cabeza y extremidades superiores. Se inició tratamiento con valproato y, posteriormente, con ACTH consiguiéndose control clínico y electroencefalográfico. Las pruebas de imagen y genéticas fueron normales. A los 11 meses de edad, comenzó con episodios paroxísticos de caída de cabeza durante el despertar, sin cambios en v-EEG con respecto a previos, y añadiéndose levetiracetam. Finalmente, se diagnosticó de espasmos infantiles sin hipsarritmia, con evolución a epilepsia con crisis mioclónicas. Dos años después, permanece asintomática y con buen desarrollo global. **Resultados**: En el primer v-EEG se observaron 10–15 descargas generalizadas en salvas, conformadas por una onda lenta hipervoltada, en ocasiones onda aguda-onda lenta, seguido de un periodo de interdescarga de desincronización generalizada de hasta 25 segundos, asociadas a hiperextensión de cabeza y MMSS y, como anomalías intercríticas, registraron brotes de ondas agudas agrupadas y complejos punta-onda de 120–300 µV de distribución multifocal. En estudios posteriores, registra incidencia leve de anomalías intercríticas, sin crisis evidentes. **Conclusiones**: El v-EEG es una prueba básica en epilepsia infantil, pues permite caracterizar la actividad encefalográfica, establecer un diagnóstico y monitorizar la respuesta farmacológica. En este caso, la clínica inicialmente era sugestiva de síndrome de West, sin embargo, el v-EEG evitó establecer un diagnostico erróneo y confirmar la efectividad del tratamiento, permitiendo un neurodesarrollo adecuado.

**19**.

**Síndrome del Túnel del Carpo Secundario a Lipofibrohamartoma**

Víctor Hugo Rubio Suárez^1^, María Concepción Maeztu Sardiña^1^, Sofía Ortigosa Gómez^1^, Reetika Mukesh Baharani Baharani^1^, Julian Vázquez Lorenzo^1^, Pilar Rosario Martínez Martínez^1^

^1^Servicio de Neurofisiología Clínica, Hospital Universitario Virgen de la Arrixaca, 30120 Murcia, España

**Introducción**: El síndrome del túnel del carpo (STC) es una patología frecuente por compresión del nervio mediano generando múltiples síntomas en el territorio inervado. Muy ocasionalmente la compresión está causada por tumoraciones benignas, como el lipofibrohamartoma (LFH), esta seudotumoración puede afectar a cualquier nervio periférico. Está constituido por tejidos adiposo y fibroso entrelazados de manera desorganizada entre las fibras nerviosas, comprimiéndolas, desplazándolas y distorsionando la anatomía intraneural. Asociado a macrodactilia, síndromes de Proteus y Klippel-Trenaunay-Weber. El diagnóstico es clínico y mediante resonancia magnética (RM). El tratamiento es la liberación simple del nervio mediano en el túnel carpiano precozmente, para evitar la degeneración walleriana. Material y método: Paciente de 4 años, origen chino, adoptado, con hipertrofia de mano derecha, de predominio en eminencia tenar y 1^∘^ dedo, al parecer no congénita, detectada al año de vida, sin otros antecedentes conocidos. A las 4 años comenzó con dolor intenso en mano derecha. Remitido por su pediatra se realizó como primera prueba diagnóstica una electromiografía (EMG), y posteriormente estudios de imagen y genéticos. **Resultados**: En la EMG, se objetivó un severo incremento de la latencia distal motora, ausencia de potenciales sensitivos distales de 2^∘^ y 3^∘^ dedo, reducción de la amplitud del 1^∘^ dedo en muñeca y ausencia de potencial mixto en palma-muñeca de nervio mediano derecho, sugestivo de atrapamiento de nervio mediano derecho en carpo de grado severo. En la RM se objetivó un lipofibrohamartoma en nervio mediano derecho. Se realizó liberación del nervio mediante cirugía. Los estudios genéticos fueron normales. **Conclusiones**: Aunque el STC es una patología común, generalmente de origen osteo-articular/tendinoso, en raras ocasiones la causa es una tumoración como la aquí descrita, que se manifiestan con síntomas de atrapamiento de nervio mediano, en un niño. El estudio EMG, permitió orientar el diagnostico hacia la búsqueda de una causa compresiva local del nervio mediano.

**20**.

**Polisomnografia de 20 Horas Positiva Después de un Test de Latencias Múltiples Negativo**

Ana Paola Salvatella-Gutiérrez^1^, Andrea Aliaga-Díaz^1^, Teresa Canet-Sanz^1^, Alba Sánchez-Tudela^1^, Lucia Soto-Manzano^1^

^1^Departamento de Neurofisiología Clínica, Hospital General Universitario Dr. Balmis, 03010 Alicante, España

**Introducción**: Los pacientes con hipersomnia idiopática pueden tener un tiempo total de sueño alargado (10 a 11 horas) además de siestas largas durante el día. El tiempo de sueño alargado medido por una polisomnografía alargada podría tener una mejor sensibilidad en pacientes con hipersomnolencia idiopática. **Caso Clínico**: Mujer de 28 años de edad con antecedente personal de hipotiroidismo y síndrome de colon irritable cuya queja principal es la hipersomnolencia diurna excesiva. Tiene hipersomnolencia desde la infancia con dificultad para despertarse por la mañana. Puede dormir un día entero y aún así sentirse cansada. Refiere parálisis del sueño al despertar del sueño nocturno y también en siestas; 1 vez por semana, también algún episodio de alucinación hipnopómpica (ver moho en las paredes). No presenta cataplejia. **Resultados**: Se realizó una polisomnografía alargada posterior a un test de latencias múltiples con resultado negativo con un tiempo total de sueño de 18 horas. **Conclusiones**: Estos estudios proporcionan una precisión y confiabilidad diagnóstica mejorada. Sin embargo, rara vez son factibles y comúnmente no están disponibles para propósitos diagnósticos.

**21**.

**Síndrome de Poems: Dos Casos Similares en dos Hospitales Diferentes**

Claudia Collado Andrés^1^, Andrés García Verdú^2^, Silvia Parra Escorihuela^1^, Victoria Cortés Doñate^3^, Alina Denisa Ghinea Nuta^1^, María Tárrega Martí^3^

^1^Servicio de Neurofisiología Clínica, Hospital General Universitario de Castellón, 12004 Castelló, España

^2^Servicio de Neurofisiología Clínica, Hospital General de Valencia, 46014 Valencia, España

^3^Servicio de Neurofisiología Clínica, Hospital La Fe de Valencia, 46026 Valencia, España

**Introducción**: El síndrome de POEMS es un acrónimo para Polineuropatía, Organomegalia, Endocrinopatía o Edema, gammapatía Monoclonal y lesiones de la piel (“Skin”). Es una enfermedad paraneoplásica muy rara que afecta a varios sistemas. La polineuropatía es una forma común de debut, que puede anteceder al resto de síntomas, por lo que puede confundirse fácilmente con una polineuropatía inflamatoria crónica desmielinizante (CIDP). Avanza rápidamente sin tratamiento y puede poner en riesgo la vida, por lo que el diagnóstico temprano es muy importante. **Objetivo**: Presentar dos casos similares de Síndrome de POEMS en dos hospitales distintos, Hospital La Fe y Hospital General de Castellón. **Casos**: Mujer de 63 años con antecedente de plasmocitoma óseo vertebral con déficit motor y sensitivo grave en MMII de varios meses de evolución, asociado a hipotiroidismo y edemas. - Varón de 30 años, con obesidad mórbida y lesiones óseas líticas a estudio, que desarrolla un déficit motor y sensitivo grave en MMII de rápida evolución con edemas. Posteriormente al diagnóstico electrofisiológico se detectó pico monoclonal y aparecieron lesiones cutáneas. - El estudio neurofisiológico en ambos pacientes mostró ausencia de conducciones sensitivas y motoras distales en MMII, con escasa afectación en MMSS (signos de desmielinización uniforme con mayor afectación de segmentos intermedios). **Discusión**: Ante un paciente que presenta clínica de polineuropatía rápidamente progresiva con afectación axonal muy grave en MMII y escasa afectación en MMSS, con desmielinización uniforme de predominio en segmentos intermedios, debemos plantear como diagnóstico diferencial el síndrome de POEMS y buscar el resto de características. Compartir casos complejos entre compañeros es beneficioso para el paciente y el profesional.

**22**.

**Coma con Patrón de Tipo Brote-Supresión y Arreflexia Troncoencefálica Secundario a Intoxicación por Baclofeno: A Propósito de un Caso**

Alba Sánchez Tudela^1^, Beatriz Romero Neva^2^, Pedro Orizaola Balaguer^2^, José Luis Fernández Torre^2^

^1^Servicio de Neurofisiología Clínica Hospital General Universitario de Alicante Dr. Balmis, 03010 Alicante, España

^2^Servicio de Neurofisiología Clínica, Hospital Universitario Marqués de Valdecilla, 39008 Santander, Cantabria, España

**Introducción**: Las causas más frecuentes del patrón electroencefalográfico de tipo brote-supresión (BS) incluyen la encefalopatía hipóxico-isquémica, sedación o anestesia profunda e hipotermia severa. Describimos un paciente en coma profundo con un patrón de tipo BS secundario a intoxicación aguda por baclofeno. **Material y Métodos**: Mujer de 63 años, con antecedentes de paraparesia secundaria a esclerosis múltiple primariamente progresiva, trastorno adaptativo mixto e intento autolítico en 2019. Su tratamiento habitual incluía mirtazapina, clonazepam, baclofeno y almotriptán. Ingresa en la Unidad de Cuidados Intensivos por un cuadro brusco de disminución del nivel de conciencia. En la exploración neurológica, la paciente se encuentra en coma profundo, con una escala de Glasgow de coma de 3, pupilas medias arreactivas y ausencia de reflejos troncoencefálicos. Los estudios de laboratorio y la Tomografía Computarizada (TC) craneal con perfusión y Angio-TC fueron normales. Se solicitó un vídeo-electroencefalograma (v-EEG) urgente. **Resultados**: En el estudio v-EEG, se observó una actividad cerebral discontinua constituida por brotes generalizados de ondas theta-delta irregulares, y periodos de supresión de la actividad cerebral configurando un patrón de tipo BS arreactivo. Se procedió a la inyección intravenosa de 1 mg de flumazenilo, objetivándose un incremento de la duración de los brotes y reducción de las fases de supresión compatibles con el diagnóstico de encefalopatía tóxico-medicamentosa severa. Posteriormente, su marido confirmó que la paciente había ingerido una cantidad tóxica de baclofeno. Al tercer día de ingreso se observó una recuperación neurológica completa. **Conclusiones**: La intoxicación por baclofeno puede ser causa de coma profundo con ausencia de reflejos troncoencefálicos y patrón de tipo BS en el v-EEG.

**23**.

**Déficit de Novo Tras Descompresión Medular**

Andrea Aliaga Díaz^1^, Elena Francés Jiménez^1^, Ana Paola Salvatella Gutiérrez^1^, Alba Sánchez Tudela^1^, Lucía Soto Manzano^1^, Teresa Canet Sanz^1^

^1^Servicio de Neurofisiología Clínica, Hospital Universitario Dr. Balmis, 03010 Alicante, España

**Introducción**: La aparición de un déficit posoperatorio tras una descompresión medular es un evento inusual, que puede deberse a diversos factores, como traumatismo medular, hematoma o descompresión inadecuada. Otra causa menos descrita en la literatura es el síndrome de reperfusión medular. Aunque su mecanismo de acción no se ha determinado, se sugiere que la rápida reperfusión tras una cirugía descompresiva provoca la generación de moléculas potencialmente dañinas. **Material y Métodos**: Varón de 42 años con lumbalgia bilateral irradiada a muslo izquierdo de 3 semanas de evolución, sin mejoría con tratamiento antiinflamatorio. Asocia episodios de incontinencia esfinteriana y disfunción eréctil. En la exploración física presenta nivel medular T5–T7, con hipoestesia y balance motor 4/5 en MII. Se realiza RM de columna que muestra estenosis multifactorial de canal a nivel D2/D3 con mielopatía compresiva, por lo que se programa para intervención quirúrgica. **Resultados**: En la Monitorización neurofisiológica intraoperatoria, presenta afectación en las respuestas basales de PEM MII, así como de PESS de ambos MMII, con indemnidad del resto de vías estudiadas. Durante la descompresión se produce una pérdida bilateral de PESS MMII y PEM MID. Al finalizar la cirugía se objetiva paresia en MID de nueva aparición. Se realiza RM de columna que muestra persistencia de la estenosis craneal a la laminectomía realizada. Se reinterviene al paciente, observándose en la MION basal una reaparición en los PESS de MMII y PEM de MID, de reducida amplitud, que se mantienen sin cambios al finalizar la cirugía. Tras la intervención, persiste paresia en MMII, de predominio derecho, con mejoría motora progresiva al alta. **Discusión**: A pesar de presentar una descompresión medular insuficiente inicialmente, la aparición de un déficit contralateral, así como la evolución clínica y neurofisiológica, nos hace considerar el síndrome de reperfusión medular como posible adyuvante del cuadro evidenciado en nuestro paciente.

**24**.

**Trastorno por Control de Impulsos en el Síndrome de Piernas Inquietas**

Andrea Aliaga Díaz^1^, Teresa Canet Sanz^1^, Sheila Picorelli Ruiz^1^, Sara Giménez Roca^1^, Alba Sánchez Tudela^1^, Ana Paola Salvatella Gutiérrez^1^

^1^Servicio de Neurofisiología Clínica, Hospital General Universitario Dr. Balmis, 03010 Alicante, España

**Introducción**: Los agonistas dopaminérgicos (AD) son frecuentemente empleados en el síndrome de piernas inquietas (SPI). Estos fármacos pueden presentar como efecto adverso notable el trastorno por control de impulsos. En nuestra Unidad del Sueño se han identificado 3 pacientes con esta patología. **Material y Métodos**: Se trata de tres varones diagnosticados de SPI, con edad media de 53 ± 1,73. En el primero se inicia Ropirinol retard 2 mg por intolerancia a Gabapentina, apareciendo incremento del impulso sexual. El segundo caso sigue tratamiento con Pramipexol 0,36 mg hace más de 20 años, con mal control sintomático, por lo que es remitido a nuestra consulta. Se diagnostica fenómeno de augmentation y se identifica incremento del impulso sexual secundario. El tercer paciente lleva 3 años en tratamiento con Rotigotina 6 mg, incrementado por persistencia de SPI, con aparición 4 meses antes de adicción a las compras compulsivas y al juego online, por lo que se remite a nuestra unidad. **Resultados**: En el primer paciente se disminuye dosis de Ropirinol hasta 0,5 mg, permaneciendo sintomatología SPI leve y desapareciendo impulsividad sexual. En el segundo caso se sustituye Pramipexol 0,36 mg por Gabapentina progresivamente, precisando dosis de 900 mg para control de sintomatología, con resolución de la impulsividad sexual. En el tercer paciente se inicia Pregabalina hasta dosis 200 mg y se disminuye Rotigotina, siendo necesario 1 mg para control de la sintomatología, pero resolviéndose impulsividad. **Conclusiones**: El trastorno por control de impulsos es un efecto secundario relevante del tratamiento AD en el SPI. Debemos indagar en su aparición con el objetivo de finalizar el tratamiento en caso de aparición de esta conducta, evitando así las consecuencias devastadoras que puede ocasionar. En dos de los casos presentados, la disminución de dosis de los AD fue suficiente para corregir la conducta compulsiva.

